# Whole Transcriptome Analysis Using Next-Generation Sequencing of Sterile-Cultured *Eisenia andrei* for Immune System Research

**DOI:** 10.1371/journal.pone.0118587

**Published:** 2015-02-23

**Authors:** Yoshikazu Mikami, Atsushi Fukushima, Takao Kuwada-Kusunose, Tetsuya Sakurai, Taiichi Kitano, Yusuke Komiyama, Takashi Iwase, Kazuo Komiyama

**Affiliations:** 1 Department of Pathology, Nihon University School of Dentistry, 1-8-13, Kanda-Surugadai, Chiyoda-ku, Tokyo 101-8310, Japan; 2 RIKEN Center for Sustainable Resource Science, 1-7-22, Suehiro, Tsurumi, Yokohama, Kanagawa 230-0045, Japan; 3 Department of Liberal Arts (Chemistry), Nihon University School of Dentistry at Matsudo, 2-870-1, Sakaecho-Nishi, Matsudo, Chiba 271-8587, Japan; 4 Intensive Care Unit, The University of Tokyo Hospital, 7-3-1 Hongo, Bunkyo-ku, Tokyo, 113-0033, Japan; University of Hong Kong, CHINA

## Abstract

Recently, earthworms have become a useful model for research into the immune system, and it is expected that results obtained using this model will shed light on the sophisticated vertebrate immune system and the evolution of the immune response, and additionally help identify new biomolecules with therapeutic applications. However, for earthworms to be used as a genetic model of the invertebrate immune system, basic molecular and genetic resources, such as an expressed sequence tag (EST) database, must be developed for this organism. Next-generation sequencing technologies have generated EST libraries by RNA-seq in many model species. In this study, we used Illumina RNA-sequence technology to perform a comprehensive transcriptome analysis using an RNA sample pooled from sterile-cultured *Eisenia andrei*. All clean reads were assembled *de novo* into 41,423 unigenes using the Trinity program. Using this transcriptome data, we performed BLAST analysis against the GenBank non-redundant (NR) database and obtained a total of 12,285 significant BLAST hits. Furthermore, gene ontology (GO) analysis assigned 78 unigenes to 24 immune class GO terms. In addition, we detected a unigene with high similarity to beta-1,3-glucuronyltransferase 1 (GlcAT-P), which mediates a glucuronyl transfer reaction during the biosynthesis of the carbohydrate epitope HNK-1 (human natural killer-1, also known as CD57), a marker of NK cells. The identified transcripts will be used to facilitate future research into the immune system using *E. andrei*.

## Introduction

Earthworms are well known as a key organism in the study of environmental toxicology [1–3]. Because they are easy to maintain in a laboratory setting [4,5], a large number of studies have been conducted using earthworms. Two earthworm species, *Eisenia fetida* and *Eisenia andrei*, have been used in European pesticide marketing authorization experiments since the early 1980s [6]. In these studies, several bioactive proteins were identified in the coelomic fluid of earthworms [7–9]. These proteins exhibit a variety of antibacterial, hemolytic, cytotoxic, hemagglutinating, and proteolytic activities [10]. Some reports have suggested that coelomocytes in the coelomic fluid play a role in immune defense [11–13]. For example, it was demonstrated that some coelomocytes possess cytotoxic activity similar to natural killer (NK) cells by co-culture with K592 cells, a human NK-sensitive cell line [14]. In that study, when the coelomocytes were co-cultured with K592 cells, they extended numerous small pseudopodia that bound to and killed K592 cells. In addition, cross-reactivity of coelomocytes with monoclonal antibodies against several human CD markers of immune-related cells was also reported [15]. Thus, earthworms are ideal models for conducting immune system research. Knowledge of the apparently less complex invertebrate immune system can contribute to our understanding of the more sophisticated vertebrate immune system, the evolution of the immune response and potentially lead to the identification of new biomolecules with therapeutic use [10,16]. However, as earthworms have only recently gained attention as a genetic model for immune system research, many basic molecular and genetic resources, such as expressed sequence tag (EST) databases, have yet to be established. EST information is an important resource for genetic and genomic studies, such as gene identification, verification of gene prediction, and gene sequence determination [17]. Traditional EST libraries are usually generated through the construction of an EST library and sequencing, which is time-consuming and expensive. Recently, with the development of the next-generation sequencing technologies, EST libraries have been successfully constructed from total RNAs in many model species [18–20]. Gui [21] analyzed the whole transcriptome of the Indo-Pacific humpback dolphin leukocyte, which resulted in the identification of putative genes involved in aquatic adaptation and the immune response. In addition, Xu [22] performed *de novo* assembly of the transcriptome of *Setaria viridis*, an emerging model species for genetic studies of C4 photosynthesis, and identified 60,751 transcripts. Their study is expected to provide the basis for future genetic studies of C4 photosynthesis. Thus, to support immune system research in earthworms, we used Illumina RNA-seq technology to perform a comprehensive transcriptome analysis using RNA samples pooled from sterile-cultured *E. andrei*. This report presents a description of the identified genes expressed in *E. andrei* and their functional annotation. All clean reads were assembled *de novo* using the Trinity program into 41,423 unigenes. Using this transcriptome data, we performed BLAST analysis against the GenBank non-redundant (NR) database and obtained a total of 12,285 significant BLAST hits. Furthermore, gene ontology (GO) analysis assigned 78 unigenes to 24 immune class GO terms. These identified transcripts can be used to facilitate future immune system research using *E. andrei*.

## Materials and Methods

### *E. andrei* materials, RNA isolation, and Illumina sequencing

The egg capsules (cocoons) of *E. andrei* were purchased from KIRYU Int. Co. (Miyazaki, Japan). After rinsing with PBS containing 50 μg/ml ampicillin, the cocoons were seeded onto 100-mm disposable petri dishes (Falcon), each coated with 100 ml of 1.0% agar supplemented with 0.5 mM NaCl, 0.05 mM KCl, 0.4 mM CaCl_2_, 0.2 mM NaHCO_3_ and 50 μg/ml ampicillin. The cultures were kept at 20°C. After the eggs hatched out, the worms were fed powdered skimmed milk every 2 weeks, and grew to a length of 3–5 cm in approximately 3 month-cultures. The adult worms were immediately frozen in liquid nitrogen and stored at −80°C until use. The frozen whole worms were ground in liquid nitrogen and total RNA was prepared using the Fastpure RNA Kit (Takara Bio Inc., Otsu, Japan) according to the manufacturer’s instructions. RNA integrity was assessed by agarose gel electrophoresis and the concentration was quantified using a Nanodrop ND-1000 spectrophotometer (Thermo Fisher Scientific, Wilmington, DE, USA). The qualified RNA samples were analyzed by the Illumina Sequencing Services of Hokkaido System Science Co., Ltd. The cDNA library for sequencing was constructed as described previously [21]. Sequencing was performed on an Illumina HiSeq 2000 [Q30 = 97.55%].

### Data pre-processing, filtering, and *de novo* assembly

For transcriptome assembly, raw reads were filtered and adapter sequences, non-coding RNA (e.g., rRNA), low-quality read sequences with ambiguous bases ‘N’, and raw reads with average lengths of less than 20 bases were removed. The Trinity program [23] was utilized for d*e novo* transcriptome assembly, combining read sequences of a certain length of overlap to form longer fragments without N gaps, called contigs. These contigs were further processed for read alignment and abundance estimation with Bowtie [24] and RSEM [25], and these sequences were defined as unigenes. Calculation of unigene expression was performed using the Fragments Per kilo base of exon per Million mapped fragments (FPKM) method, which was able to exclude sequencing discrepancies in the calculation of gene expression and the influence of different gene lengths. The number of unigenes was 41,423 with a threshold of more than FPKM = 2. All raw read sequences are available at the DDBJ Sequence Read Archive [26] with the accession number: DRA002587.

### Functional annotation of unigenes and simple sequence repeats (SSR) identification

A homology search against the NCBI non-redundant (NR) protein database (http://www.ncbi.nlm.nih.gov; formatted on April 7, 2014) based on the BLASTx program [27] was performed for all unigenes using a cutoff E-value < 1E^−6^, and the best aligning results were selected to annotate the unigenes. To further annotate the unigenes, the Blast2GO program v. 2.7.1 [28] was used to obtain gene ontology (GO) [29] terms and the Kyoto Encyclopedia of Genes and Genomes (KEGG) [30] information according to the NR results. The BGI WEGO program [31] was used to perform GO functional classification of all unigenes to visualize the distribution of gene functions. The identification of immune-related genes was performed as described by Pereiro et al. [32]. Furthermore, to detect more genes belonging to the relevant immune pathways in the transcriptome sequences, KEGG reference pathways identified in a previous report were utilized [21]. Microsatellite identification tool (MISA) (http://pgrc.ipk-gatersleben.de/misa/) with default parameters was used to detect microsatellites in the unigenes.

### Prediction of the tertiary structure of *E. andrei* GlcAT-P

The tertiary structure of *E. andrei* GlcAT-P was predicted by homology modeling with SWISS-MODEL server [33]. In the homology modeling, the GlcAT-P structure from human (Protein Data Bank (PDB) entry 1v83) [34] was used as a template. Structural Figs. were drawn using PyMOL [35].

## Results and Discussion

### Illumina sequencing and sequence assembly

**Fig 1:**
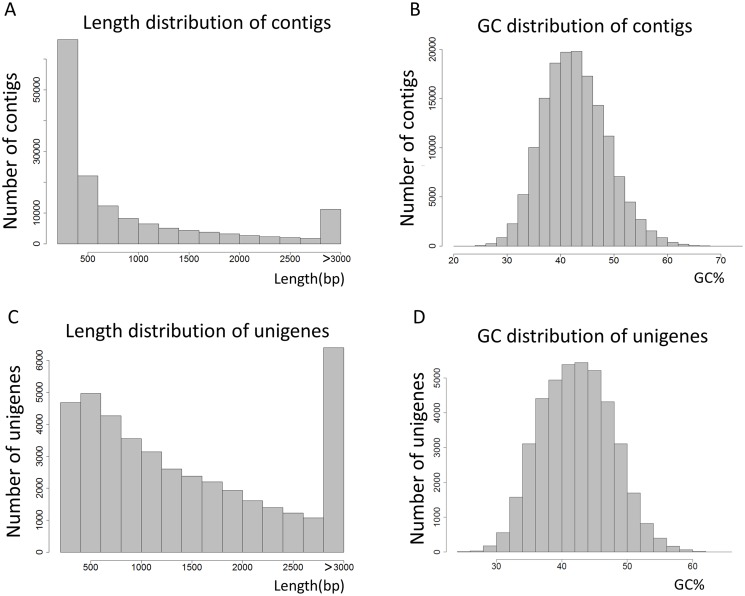
Overview of *E. andrei* transcriptome assembly. (A and B) The lengths and GC distributions of the contigs obtained from *de novo* assembly of high-quality clean reads. (C and D) The lengths and GC distributions of the unigenes produced from further contig assembly.

In total, Illumina sequencing yielded 47,555,164 reads comprising 4,803 M bases from the mRNA pool isolated from sterile-cultured *E. andrei*. After the removal of adaptor sequences, ambiguous reads and low-quality reads, we obtained 47,070,010 clean reads. The Q30 percentage (sequencing error rate, 0.01%) was 97.55%. As shown in Table 1, 47,070,010 clean reads were assembled *de novo* using the Trinity program. The clean reads were further assembled into 151,929 contigs with an average length of 968 bp and an N50 of 1,855 bp. The length and GC content distribution for all contigs are shown in Fig. 1A and 1B. In total, 72,539 contigs were longer than 500 bp and the GC of 17,320 contigs was greater than 50%. From the contigs, 41,423 unigenes were obtained with an average length of 1,633 bp and an N50 of 1,567 bp (Table 1). The length and GC distributions of all assembled unigenes are shown in Fig. 1C and 1D. In total, 7,219 unigenes were less than 500 bp and 5,488 unigenes were longer than 3,000 bp. The GC content of 4,722 unigenes was greater than 50%.

**Table 1:** Summary of the sequence assembly after Illumina sequencing.

	Total Number	Total length (bp)	Average length (bp)	Average GC (%)	N50 (bp)	Q30 (%)
Raw sequencing reads	47,555,164	4,803,000,000				97.55
Total clean reads	47,070,010					
Total contigs	151,929	147,023,550	968	42.7	1,855	
Total unigenes	41423	76,015,064	1,633	42.8	1,567	

### Functional annotation

**Fig 2:**
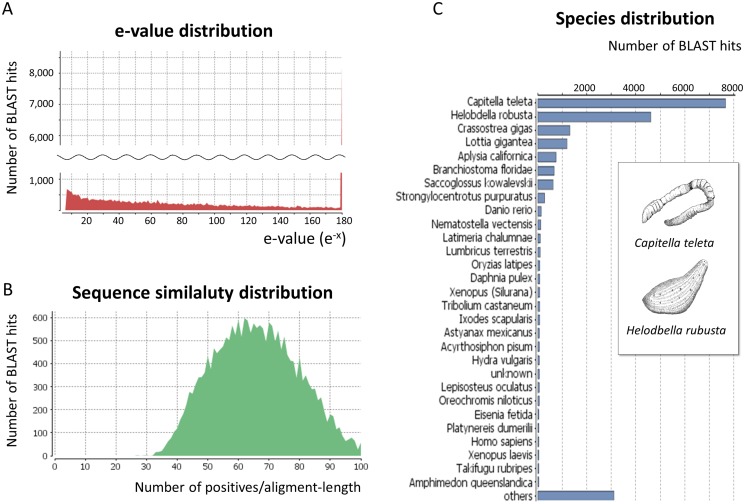
Characterization of the assembled unigenes based on an NR protein database search. (A) E-value distribution of BLAST hits for the assembled unigenes with a cutoff of E-value < 1E^-6^. (B) Similarity distribution of the top BLAST hits for the assembled unigenes with a cutoff of E-value < 1E^-6^. (C) Species distribution of the top BLAST hits for the assembled unigenes with a cutoff of E-value < 1E^-6^.

To validate and annotate the assembled unigenes, all assembled unigenes were searched against the GenBank non-redundant (NR) database using the BLASTx program (E-value < 1E^-6^). The results showed that 12,285 unigene sequences had BLAST hits to annotated proteins in NR. Analysis of the distributions of E-values indicated that 82.7% of the aligned sequences showed significant homology to the entries in the NR database (E-value < 1E^-6^) (Fig. 2A). Further analysis of the similarity distributions indicated that 73.3% of matched sequences had alignment identities greater than 80% (Fig. 2B). A large number of the hits matched the sequences of *Capitella teleta* (24.8%) and *Helobdella robusta* (18.1%); other hits were identified within the reference protein databases of *Crassostrea gigas* (7.3%), *Lottia gigantea* (5.7%), *Aplysia californica* (5.4%), *Saccoglossus kowalevskii* (4.8%), and *Strongylocentrotus purpuratus* (4.7%) (Fig. 2C). There were also many unigenes with no BLAST hits; these might represent additional genes that were not represented in the annotated protein databases or sequences that were too short to produce hits.

**Fig 3:**
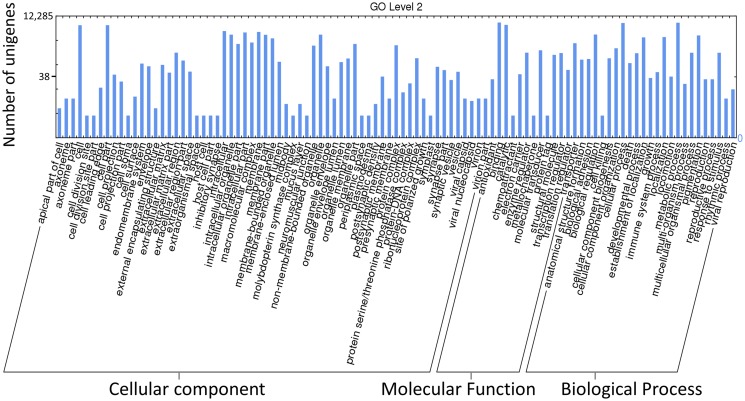
GO assignments for assembled unigenes. The results are summarized in three main categories: biological process, cellular component, and molecular function. In total, 12,285 unigenes were categorized by GO term. Classified gene objects are depicted as absolute numbers of the total number of gene objects with GO assignments.

GO (gene ontology) is an international system for the classification of standardized gene functions and is used to annotate and analyze gene functions and gene products in any organism. GO contains three main independent ontologies: biological process, molecular function, and cellular component. To predict their possible functions, we used the Blast2GO program to analyze the GO annotations of the assembled unigenes, and then used WEGO software to perform GO functional classifications. Based on NR annotation, 12,285 unigenes were assigned to 99 GO terms belonging to the three main GO ontologies (Fig. 3). Further analysis of the 99 GO terms showed that the dominant terms were “cell”, “cell part”, “binding”, “catalytic”, “cellular process”, and “metabolic process”. Within the biological process group, the great majority of unigenes were related to the terms “cellular process” and “metabolic process”. Within the cellular component, most unigenes were assigned to “cells” and “cell parts”, followed by “binding” and “catalytic activity”.

**Fig 4:**
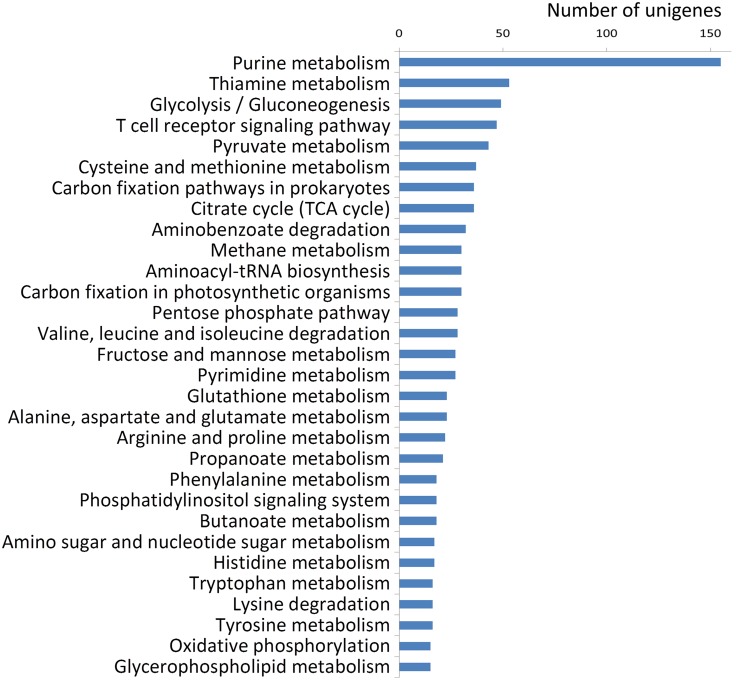
Pathway enrichment analysis of assembled unigenes. In total, 1,322 unigenes were grouped into 100 KEGG pathways. The top 30 pathways containing unigenes are shown.

KEGG, a pathway-based categorization of orthologous genes, provides useful information for predicting the functional profiles of genes. Therefore, to better understand the function of the *E. andrei* transcriptome, the unigenes were assigned against the KEGG pathway. In total, 1,322 unigenes were grouped into 100 KEGG pathways (S1 Table). The top three ranking pathways were purine metabolism (155 unigenes), thiamine metabolism (53 unigenes), and glycolysis/gluconeogenesis (49 unigenes) (Fig. 4). Interestingly, 47 unigenes mapped to the T cell receptor signaling pathway, which is involved in immune response (Fig. 4).

### SSR markers identification

Microsatellites, also known as SSRs, are classes of repetitive DNA sequences ubiquitous in eukaryotic genomes [36]. SSRs are ideal markers for use in paternity determination, population genetics studies, genetic diversity assessment and genetic map development [37]. The results of microsatellite analysis are shown in Table 2 and S2 Table. From the unigene sequences, 14,717 SSRs (10,764 with simple repeats and 722 with compound formation) were identified in 12,539 unique sequences, of which, 1,775 sequences contained more than one SSR. All SSRs were further classified by the number of repeat units. The results showed that the number of most potential SSRs were composed of three repeat units (54.1%), followed by two (31.2%), four (7.7%), and four (6.8%) repeat units. The SSRs of *E. andrei* could provide potential genetic markers for studies of population genetics, comparative genomics, as well as gene-based association studies aimed at understanding the genetic control of adaptive traits.

**Table 2:** Number of SSRs identified in the transcriptome of *E. andrei*.

Total number of identified SSRs:	14717
Number of SSR containing sequence:	12539
Number of sequences containing more than one SSR:	1775

Distribution to different repeat type classes			
Unit size	Number of SSRs	Percentage of SSRs
1	1007	6.8
2	4591	31.2
3	7969	54.1
4	1134	7.7
5	16	0.1

### Identification of sequences related to the immune response

**Fig 5:**
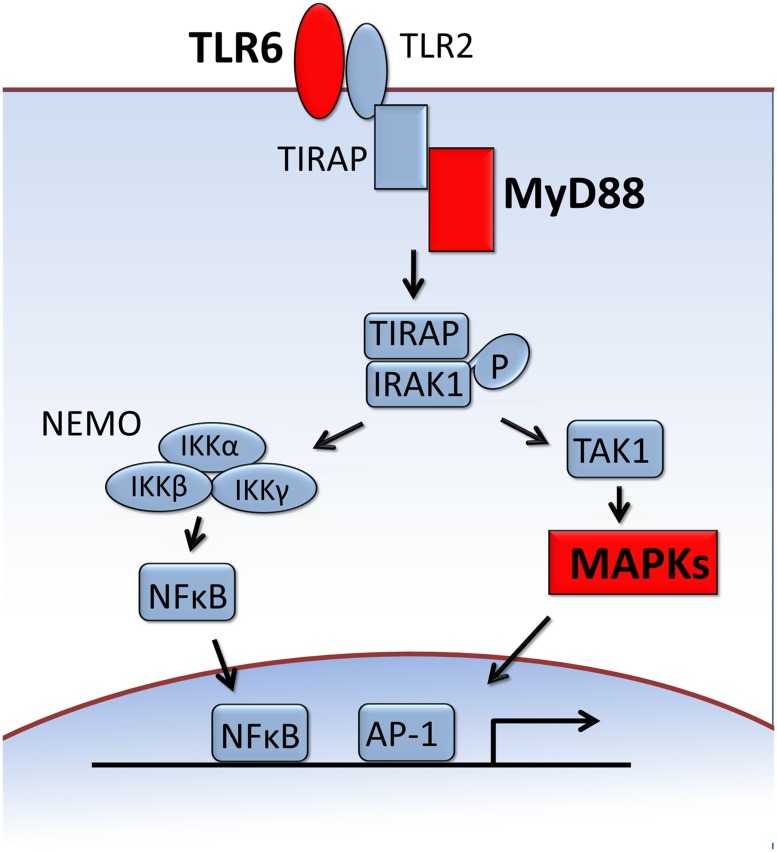
TLR signaling pathway. The red boxes indicate BLAST hits against the NR protein database.

A keyword list and GO immune-related terms were used to search for genes putatively involved in the immune system of *E. andrei* (S3 Table). The results of this search identified a number of immune-related genes that were involved in well-recognized immune pathways, such as toll-like receptor (TLR) and interferon-gamma-mediated signaling. The toll receptor, as the signal transducer of the Toll pathway, plays a crucial role in the innate immune response. In this study, we identified a gene encoding TLR6 in the transcriptome datasets, as well as other genes belonging to the TLR signaling pathway, such as myeloid differentiation primary response protein (MyD) 88 and mitogen-activated protein kinases (MAPKs) [Fig. 5]. Members of the JAK (Janus kinase) family are intracellular, non-receptor tyrosine kinases that transduce cytokine-mediated signals via the JAK-STAT pathway [38]. Many studies have shown that signal transducers and activators of transcription (STAT) proteins are involved in the development and function of the immune system and play a role in maintaining immune tolerance and tumor surveillance [39]. In our study, a unigene was identified with high similarity to mammalian JAK 2 but not to other members of the STAT family (STAT1, STAT2, STAT3, STAT4, STAT5A/5B, and STAT6). Identification of additional JAK-STAT pathway-related genes will be useful for learning more about the complexities of immune responses in *E. andrei*. In addition, signaling and interaction molecules, such as cytokines and cytokine receptors, were also identified in the transcriptome, as were proteases, protease inhibitors, and stress proteins such as heat shock proteins.

### Identification of markers of NK cell

**Fig 6:**
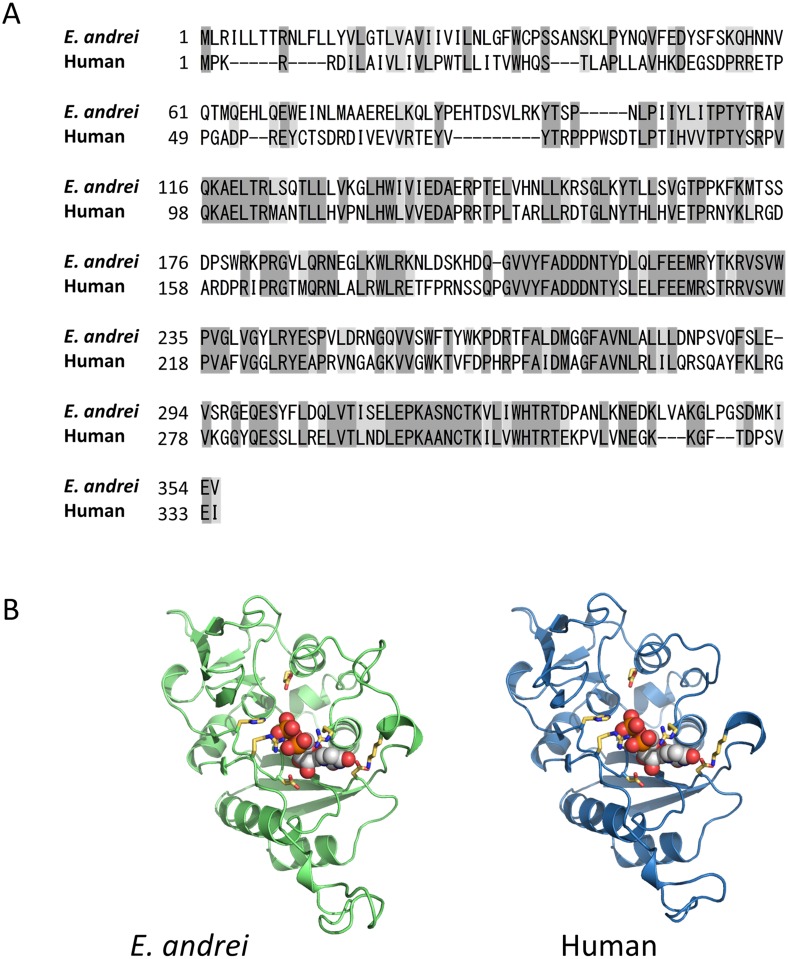
Identification of *E. andrei* GlcAT-P. (A) Comparison of the amino acid sequences of human and *E. andrei* GlcAT-P proteins. Amino acid sequences are shown as a single-letter code. Amino acids that are conserved between human and *A. andrei* are shaded. (B) The predicted tertiary structure of *E. andrei* GlcAT-P. The tertiary structure of *E. andrei* GlcAT-P was modeled using the structure of human GlcAT-P as a template. In each GlcAT-P structure, UDP binding regions and amino acid residues in the active site are shown as a ball and stick models, respectively. The position of the UDP binding region in *E. andrei* GlcAT-P is based on the human GlcAT-P structure.

NK cells are a type of cytotoxic lymphocyte that plays an important role in the innate immune system. The development of NK cells was a critical event in the evolution of the immune system [40]. Despite their importance, the evolutionary origin of NK cells has remained unknown [41]. Previous reports have suggested that earthworms have NK-like cells [12–14]. Therefore, these animals may occupy a key position in the evolution of the NK cell-dependent immune system. Thus, we next searched our unigene database for markers of NK cells, such as CD16 and CD56, and NK-related genes, using the BLASTx program (E-value < 1E^-6^). As shown in Fig. 6A, we detected a unigene with high similarity to beta-1,3-glucuronyltransferase 1 (GlcAT-P), which mediates a glucuronyl transfer reaction during biosynthesis of the carbohydrate epitope HNK-1 (human natural killer-1, also known as CD57, an NK cell marker). The similarity was much higher than that of other unigenes to human NK cell markers. Thus, we further investigated this unigene. We found that the unigene has 44% amino acid sequence identity and 55% amino acid sequence similarity with human GlcAT-P. The structure of human GlcAT-P and the structural basis for acceptor substrate recognition of human GlcAT-P in the biosynthesis of HNK-1 have been reported [30]. However, because newly synthesized polypeptide chains must undergo folding into tertiary conformations and post-translational modifications before becoming a functional protein, a high level of identity in primary amino acid sequence between species does not automatically confer identical tertiary structure. Thus, as a preliminary analysis of the tertiary structure, we estimated the structure of earthworm GlcAT-P using homology modeling with the human GlcAT-P, and obtained the tertiary structure of amino acid residues 101–355 of *E. andrei* GlcAT-P, including the uridine diphosphate (UDP) binding region which plays an important role in enzyme activity of GlcAT-P. As shown in Fig. 6B, S1 and S2 Movies, a high level of conservation of the UDP binding region was predicted between these two species in the tertiary structures of GlcAT-P. This suggests that the carbohydrate epitope HNK-1 is synthesized in *E. andrei* and that an anti-CD57 antibody could be used to identify NK-like cells in *E. andrei*.

Epigeic species of earthworms, including *E. andrei*, live in top layer of the soil, which is rich in decaying organic matter and characterized by a wide variety of microbiota. These environmental contaminants and microbiota, including viruses, bacteria, and protozoans, could interfere with the abundance of epigeic earthworms by inducing a high mortality rate, decreasing reproductive success or synergistically increasing the virulence of other diseases. Therefore, to be successful as an epigeic earthworm, *E. andrei* needs to be resistant to various microorganisms present in the top layer of the soil. However, the mechanisms responsible for immune responses to these environmental contaminants and microbiota in *E. andrei* are not fully understood. Innate immunity is the first line of host defense against pathogens. Many immune cells, such as monocytes, macrophages, leukocytes (PMN), and NK cells, are involved in the detection and removal of microbial pathogens [42,43]. Compared to other model organisms such as mice and fruit flies, few immune-related genes have been identified in *E. andrei*. Our results revealed a large number of innate immune-related genes, covering almost all known innate immune pathways, including pathogen recognition, modulation, and signaling. These findings will facilitate our comprehensive understanding of the mechanisms involved in the immune response to environmental contaminants and microbiota in *E. andrei*.

In conclusion, we characterized the transcriptome of *E. andrei* and identified many SSRs and abundant specific gene families involved in the immune response. To our knowledge, this is the first report to analyze the whole transcriptome of this epigeic species of earthworm. The dataset generated in this study will provide a substantial transcriptome-level resource for the study of earthworm biology and help further our understanding of the molecular mechanisms of various pathways, including those of the immune response.

## Supporting Information

S1 Movie Tertiary structure of human GlcAT-P. The human GlcAT-P structure was derived from Protein Data Bank (PDB) entry 1v83.
(MPG)

S2 Movie Tertiary structure of *E. andrei* GlcAT-P. The tertiary structure of *E. andrei* GlcAT-P was predicted by homology modeling with SWISS-MODEL server. In the homology modeling, the GlcAT-P structure from human was used as a template.
(MPG)

S1 Table Pathway enrichment analysis of assembled unigenes of *E. andrei*. The unigenes were assigned against the KEGG pathway.
(XLSX)

S2 Table List of SSRs detected in the unigenes of *E. andrei*. Microsatellite identification tool (MISA) with default parameters was used to detect SSRs in the unigenes.
(XLSX)

S3 Table GO terms used for identifying immune-related genes. (XLSX)

## References

[1] Spurgeon DJ, Svendsen C, Lister LJ, Hankard PK, Kille P. Earthworm responses to Cd and Cu under fluctuating environmental conditions: a comparison with results from laboratory exposures. Environ Pollut. 2005;136: 443–452. 15862398 10.1016/j.envpol.2005.01.013

[2] Gao Y, Sun X, Gu X, Sun Z. Gene expression responses in different regions of Eisenia fetida with antiparasitic albendazole exposure. Ecotoxicol Environ Saf. 2013;89: 239–244. 10.1016/j.ecoenv.2012.12.004 23290683

[3] Pauwels M, Frérot H, Souleman D, Vandenbulcke F. Using biomarkers in an evolutionary context: lessons from the analysis of biological responses of oligochaete annelids to metal exposure. Environ Pollut. 2013;179: 343–250. 10.1016/j.envpol.2013.05.005 23707006

[4] Karaka A (ed). Biology of Earthworms, Soil Biology 2011;24, Springer-Verlag Berlin Heidelberg.

[5] Salzet M, Tasiemski A, Cooper E. Innate immunity in lophotrochozoans: the annelids. Curr Pharm Des. 2006;12: 3043–3050. 16918433 10.2174/138161206777947551

[6] Pelosi C, Joimel S, Makowski D. Chemosphere. Searching for a more sensitive earthworm species to be used in pesticide homologation tests—A meta-analysis. Chemosphere. 2013;90: 895–900. 10.1016/j.chemosphere.2012.09.034 23084259

[7] Yuk J, Simpson MJ, Simpson AJ. Coelomic fluid: a complimentary biological medium to assess sub-lethal endosulfan exposure using ¹H NMR-based earthworm metabolomics. Ecotoxicology. 2012;21: 1301–1313. 10.1007/s10646-012-0884-5 22451197

[8] Cooper EL, Kvell K, Engelmann P, Nemeth P. Still waiting for the toll? Immunol Lett. 2006;104: 18–28. 16368151 10.1016/j.imlet.2005.11.012

[9] Kobayashi H, Ohta N, Umeda M. Biology of lysenin, a protein in the coelomic fluid of the earthworm *Eisenia foetida* . Int Rev Cytol. 2004;236: 45–99. 15261736 10.1016/S0074-7696(04)36002-X

[10] Salzet M, Stefano GB. The endocannabinoid system in invertebrates. Prostaglandins Leukot Essent Fatty Acids. 2002;66: 353–361. 12052049 10.1054/plef.2001.0347

[11] Cossarizza A, Cooper EL, Quaglino D, Salvioli S, Kalachnikova G, Franceschi C. Mitochondrial mass and membrane potential in coelomocytes from the earthworm *Eisenia foetida*: studies with fluorescent probes in single intact cells. Biochem Biophys Res Commun. 1995;214: 503–510. 7677758 10.1006/bbrc.1995.2315

[12] Cooper EL. Phylogeny of cytotoxicity. Endeavor. 1981;4: 160. 10.1016/0160-9327(80)90006-x 6160999

[13] Franceschi C, Cossarizza A, Monti D, Ottaviani E. Cytotoxicity and immunocyte markers in cells from the freshwater snail *Planorbarius corneus* (L.) (*Gastropoda pulmonata*): implications for the evolution of natural killer cells. Eur J Immunol. 1991;21: 489–493. 1999228 10.1002/eji.1830210235

[14] Suzuki MM, Cooper EL. Spontaneous cytotoxic earthworm leukocytes kill K562 tumor cells. Zoolog Sci. 1995;12: 443–451. 8528015 10.2108/zsj.12.443

[15] Engelmann P, Pál J, Berki T, Cooper EL, Németh P. Earthworm leukocytes react with different mammalian antigen-specific monoclonal antibodies. Zoology (Jena). 2002;105: 257–265. 16351874 10.1078/0944-2006-00068

[16] Aitlhadj L, Stürzenbaum SR. *Caenorhabditis elegans* in regenerative medicine: a simple model for a complex discipline. Drug Discov Today. 2014;19: 730–734. 10.1016/j.drudis.2014.01.014 24513577

[17] Parkinson J, Blaxter M. Expressed sequence tags: an overview. Methods Mol Biol. 2009;533: 1–12. 10.1007/978-1-60327-136-3_1 19277571

[18] Elingaramil S, Li X, He N. Applications of nanotechnology, next generation sequencing and microarrays in biomedical research. J Nanosci Nanotechnol. 2013;13: 4539–4551. 23901472 10.1166/jnn.2013.7522

[19] Wang YB, Chen SH, Lin CY, Yu JK. EST and transcriptome analysis of cephalochordate amphioxus—past, present and future. Brief Funct Genomics. 2012;11: 96–106. 10.1093/bfgp/els002 22308056

[20] Turner DJ, Keane TM, Sudbery I, Adams DJ. Next-generation sequencing of vertebrate experimental organisms. Mamm Genome. 2012;20: 327–338. 10.1007/s00335-009-9187-4 PMC2714443 19452216

[21] Gui D, Jia K, Xia J, Yang L, Chen J, Wu Y, et al De novo assembly of the Indo-Pacific humpback dolphin leucocyte transcriptome to identify putative genes involved in the aquatic adaptation and immune response. PLoS One. 2013;8: e72417 10.1371/journal.pone.0072417 24015242 PMC3756080

[22] Xu J, Li Y, Ma X, Ding J, Wang K, Wang S, et al Whole transcriptome analysis using next-generation sequencing of model species *Setaria viridis* to support C4 photosynthesis research. Plant Mol Biol. 2013;83: 77–87. 10.1007/s11103-013-0025-4 23512102

[23] Grabherr MG, Haas BJ, Yassour M, Levin JZ, Thompson DA, Amit I, et al Full-length transcriptome assembly from RNA-Seq data without a reference genome. Nat Biotechnol. 2011;29: 644–652. 10.1038/nbt.1883 21572440 PMC3571712

[24] Langmead B, Trapnell C, Pop M, Salzberg SL. Ultrafast and memory-efficient alignment of short DNA sequences to the human genome. Genome Biol. 2009;10: R25 10.1186/gb-2009-10-3-r25 19261174 PMC2690996

[25] Li B, Dewey CN. RSEM: accurate transcript quantification from RNA-Seq data with or without a reference genome. BMC Bioinformatics. 2011;12: 323 10.1186/1471-2105-12-323 21816040 PMC3163565

[26] Kosuge T, Mashima J, Kodama Y, Fujisawa T, Kaminuma E, Ogasawara O, et al DDBJ progress report: a new submission system for leading to a correct annotation. Nucleic Acids Res. 2014;42: D44–49. 10.1093/nar/gkt1066 24194602 PMC3964987

[27] Kent WJ. BLAT—the BLAST-like alignment tool. Genome Res. 2002;12: 656–664. 11932250 10.1101/gr.229202 PMC187518

[28] Conesa A, Gotz S, Garcia-Gomez JM, Terol J, Talon M, Robles M. Blast2GO: a universal tool for annotation, visualization and analysis in functional genomics research. Bioinformatics. 2005;21: 3674–3676. 16081474 10.1093/bioinformatics/bti610

[29] Gene Ontology Consortium. Creating the gene ontology resource: design and implementation. Genome Res. 2001;11: 1425–1433. 11483584 10.1101/gr.180801 PMC311077

[30] Kanehisa M, Goto S. KEGG: kyoto encyclopedia of genes and genomes. Nucleic Acids Res. 2000;28: 27–30. 10592173 10.1093/nar/28.1.27 PMC102409

[31] Ye J, Fang L, Zheng H, Zhang Y, Chen J, Zhang Z, et al WEGO: a web tool for plotting GO annotations. Nucleic Acids Res. 2006;34: W293–297. 16845012 10.1093/nar/gkl031 PMC1538768

[32] Pereiro P, Balseiro P, Romero A, Dios S, Forn-Cuni G, Fuste B, et al High-Throughput Sequence Analysis of Turbot (*Scophthalmus maximus*) Transcriptome Using 454-Pyrosequencing for the Discovery of Antiviral Immune Genes. Plos One. 2012;7: e35369 10.1371/journal.pone.0035369 22629298 PMC3356354

[33] Biasini M, Bienert S, Waterhouse A, Arnold K, Studer G, Schmidt T, et al “SWISS-MODEL: modelling protein tertiary and quaternary structure using evolutionary information. Nucleic Acids Res. 2014;42: W252–258. 10.1093/nar/gku340 24782522 PMC4086089

[34] Kakuda S, Shiba T, Ishiguro M, Tagawa H, Oka S, Kajihara Y, et al “Structural basis for acceptor substrate recognition of a human glucuronyltransferase, GlcAT-P, an enzyme critical in the biosynthesis of the carbohydrate epitope HNK-1. J Biol Chem. 2004;279: 22693–22703. 14993226 10.1074/jbc.M400622200

[35] Schrödinger LLC. The PyMOL Molecular Graphics System, Version 1.5.0.3. 2014.

[36] Toth G, Gaspari Z, Jurka J. Microsatellites in different eukaryotic genomes: survey and analysis. Genome Res. 2000;10: 967–981. 10899146 10.1101/gr.10.7.967 PMC310925

[37] Goldstein DB RLA, Cavalli-Sforza LL, Feldman MW. An evaluation of genetic distances for use with microsatellite loci. Genetics. 1995;139: 463–471. 7705647 10.1093/genetics/139.1.463 PMC1206344

[38] Seavey MM, Dobrzanski P. The many faces of Janus kinase. Biochemical Pharmaco. 2012;83: 1136–1145. 10.1016/j.bcp.2011.12.024 22209716

[39] O’Shea JJ, Plenge R. JAK and STAT Signaling Molecules in Immunoregulation and Immune-Mediated Disease. Immunity. 2012;36: 542–550. 10.1016/j.immuni.2012.03.014 22520847 PMC3499974

[40] Adams EJ, Parham P. Species-specific evolution of MHC class I genes in the higher primates. Immunol Rev. 2001;183: 41–64. 11782246 10.1034/j.1600-065x.2001.1830104.x

[41] Khalturin K, Becker M, Rinkevich B, Bosch TC. Urochordates and the origin of natural killer cells: identification of a CD94/NKR-P1-related receptor in blood cells of Botryllus. Proc Natl Acad Sci USA. 2003;100: 622–627. 12518047 10.1073/pnas.0234104100 PMC141046

[42] Matangkasombut P. Cell mediated immune response. Current concepts and approaches. J Med Assoc Thai. 1970;53: 831–835. 5498865

[43] Wilkie BN. Respiratory tract immune response to microbial pathogens. J Am Vet Med Assoc. 1982;181: 1074–1079. 6294027

